# Cancer-Specific hNQO1-Responsive Biocompatible Naphthalimides Providing a Rapid Fluorescent Turn-On with an Enhanced Enzyme Affinity

**DOI:** 10.3390/s20010053

**Published:** 2019-12-20

**Authors:** Sun Young Park, Eugeine Jung, Jong Seung Kim, Sung-Gil Chi, Min Hee Lee

**Affiliations:** 1 Department of Chemistry, Sookmyung Women’s University, Seoul 04310, Korea; 1411437@sookmyung.ac.kr; 2 Department of Life Sciences, Korea University, Seoul 02841, Korea; eugeine211@gmail.com; 3 Department of Chemistry, Korea University, Seoul 02841, Korea

**Keywords:** human NAD(P)H:quinone oxidoreductase 1 (hNQO1), fluorescent off-on naphthalimides, trimethyl lock quinone, triphenylphosphonium salt

## Abstract

Human NAD(P)H:quinone oxidoreductase 1 (hNQO1) is overexpressed in cancer cells and associated with the drug resistance factor of cancer. The objective of this work is the development of fluorescent probes for the efficient detection of hNQO1 activity in cancer cells, which can be employed for the cancer diagnosis and therapeutic agent development. Herein, we report naphthalimide-based fluorescent probes **1** and **2** that can detect hNQO1. For hNQO1 activity, the probes showed a significant fluorescence increase at 540 nm. In addition, probe **1**, the naphthalimide containing a triphenylphosphonium salt, showed an enhanced enzyme efficiency and rapid detection under a physiological condition. The detection ability of probe **1** was superior to that of other previously reported probes. Moreover, probe **1** was less cytotoxic during the cancer cell imaging and readily provided a strong fluorescence in hNQO1-overexpressed cancer cells (A549). We proposed that probe **1** can be used to detect hNQO1 expression in live cells and it will be applied to develop the diagnosis and customized treatment of hNQO1-related disease.

## 1. Introduction

Human NAD(P)H:quinone oxidoreductase 1 (hNQO1) is a flavoenzyme that has a critical role in cellular defense against oxidative stress. The hNQO1 is primarily involved in the reduction of quinones with a cofactor NAD(P)H [1]. Upregulation of hNQO1 level has been found in many cancer types, including lung, colorectal, liver, ovarian, and breast cancers [2,3,4]. The hNQO1 can reduce the effectiveness of a quinone-based anticancer drug, which interrupts drug activity and leads to cancer progression [5]. Thus, it has been suggested that hNQO1 is a potential biomarker in the diagnosis and treatment of cancer [6,7].

However, the biological roles of hNQO1 in an antioxidant defense system and cancer are not fully understood. Imaging of hNQO1 in cancer cells remains a major challenge. Recently, some fluorescent molecule-based probes that can detect hNQO1 activity in cancer cells have been reported [8,9,10,11,12,13]. Nonetheless, there are limitations such as fluorescence signals being interfered with by cellular autofluorescence and poor solubility. To overcome these limitations, we aim to develop a system that shows enhanced solubility and a high fluorescence signal at a relatively long wavelength (>500 nm) in biological milieus.

**Scheme 1:**
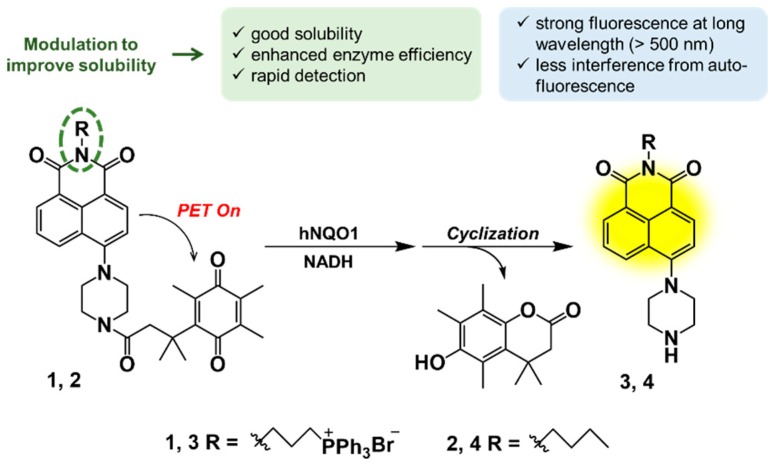
Detection mechanism of probes **1** and **2** for hNQO1 activity.

Here, we developed hNQO1-responsive fluorescent turn-on naphthalimides (probes **1** and **2**) that can provide a strong fluorescence at wavelength range of 500–650 nm. To improve the solubility of probe **1** in the biological milieus, we introduced a triphenylphosphonium group, as a cationic and lipophilic salt, on the naphthalimide-trimethyl lock quinone (TLQ) framework. In addition, we prepared probe **2** without a triphenylphosphonium moiety. Moreover, the TLQ group was employed as a hNQO1-sensitive reaction site and fluorescence quencher [8]. As illustrated in Scheme 1, towards hNQO1 activity, a TLQ group of probes **1** and **2** can be cleaved to produce highly fluorescent naphthalimides **3** and **4**.

## 2. Materials and Methods

### 2.1. Materials and Instrumentations

All reagents, hNQO1, and NADH were purchased from TCI (Tokyo, Japan), Aldrich (St. Louis, MO, USA), and Alfa (Heysham, LA3 2XY, UK) and utilized without further purification. All absorption and fluorescence spectroscopic analyses were carried out by UV-2600 (Shimadzu Corporation, Kyoto, Kyoto Prefecture, Japan) and RF-6000 (Shimadzu Corporation, Kyoto, Kyoto Prefecture, Japan) spectrophotometer, respectively. The ^1^H, ^13^C NMR were recorded with a Bruker instrument (500 MHz NMR, Bruker Corporation, Billerica, MA, USA). The purities of probes were also determined by ^1^H NMR using dimethyl sulfone as an internal standard. HR-ESI-MS analyses were carried out using liquid chromatography mass spectrometer (LC/MS) at the Korea Basic Science Institute (KBSI, Seoul, Korea). Fluorescence images were obtained using a confocal laser scanning microscope (Carl-Zeiss LSM 700 Exciter, Oberkochen, Germany).

### 2.2. Cell Culture and Confocal Microscopic Methods

A549 (human lung epithelial adenocarcinoma) and H596 (human lung adenosquamous carcinoma) cells were obtained from the Korean Cell Line Bank (Seoul, Korea). Approximately 2.5 × 10^4^/mL (total volume 2 mL) cells were seeded on glass-bottomed confocal dishes (SPL Life Sciences, Gyeonggi-do, Korea) for two days before imaging. The cells were maintained in a humidified atmosphere of 5% CO_2_ at 37 °C. The culture media for each cell-line are as below. The cell lines were grown on the same media conditions, specific compositions are as follows: RPMI 1640 (GE Healthcare, Chicago, IL, USA) supplemented with 10% fetal bovine serum (Gibco, Grand Island, NY, USA), as well as 1% penicillin and streptomycin (Gibco, Grand Island, NY, USA). The cells were incubated with 5 μM of probe for 1 h and washed three times with PBS. In confocal laser scanning microscopic experiments, the fluorescence channel was excited at 405 nm and the emission was collected by a 430–540 nm band-pass filter. Magnification of each image was 40×.

### 2.3. Synthesis

Probes **1** and **2** were synthesized as shown in Scheme 2. Compounds **5** [14], **6** [15] and **7** [16] were prepared by using the literature procedures. Chemical structure of synthesized compounds **1**, **2**, **3**, and **4**, were confirmed by ^1^H, ^13^C NMR spectroscopy and high-resolution mass spectrometry (Figures S17–S28).

#### 2.3.1. Synthesis of Probe **1**

Triphenylphosphonium-naphthalimide-piperazine **3** (200 mg, 1.0 mmol) was treated with *N*-(3-dimethylaminopropyl)-*N′*-ethylcarbodiimide hydrochloride (EDC) (130 mg, 2.0 mmol) and 4-dimethylaminopyridine (DMAP) (125 mg, 3.0 mmol) in DMF (5 mL) under N_2_ atmosphere, and stirred for 30 min. Quinone **7** (97 mg, 1.0 mmol) was then added to a mixture, and stirred at room temperature for 14 h. The reaction completion was monitored by using a silica gel-coated TLC plate. The reaction mixture was diluted with CH_2_Cl_2_ (100 mL), and washed with water (100 mL). The organic layer was collected, dried over anhydrous Na_2_SO_4_, and concentrated in vacuo. The resulting crude extract was purified by silica gel column chromatography (CH_2_Cl_2_:MeOH = 20:1), to give a yellow solid **1** (90 mg, 32%). HR–ESI–MS *m*/*z* [M]^+^ calc. 816.3561, [M]^+^ obs. 816.3558 (mass error = −0.29 ppm). Purity was determined by ^1^H NMR: 95%; ^1^H NMR (DMSO-*d*_6_, 500 MHz): *δ* 8.58–8.57 (d, *J* = 8.0 Hz, 1H), 8.53–8.51 (d, *J* = 7.5 Hz, 1H), 8.45–8.43 (d, *J* = 8.0 Hz, 1H), 7.96–7.93 (m, 3H), 7.90–7.82 (m, 16H), 7.41–7.40 (d, *J* = 8.5 Hz, 1H), 4.27–4.26 (m, 2H), 3.86–3.76 (m, 6H), 3.26–3.15 (m, 4H), 3.11 (s, 2H), 2.10 (s, 3H), 2.05–2.01 (m, 2H), 1.93 (s, 3H), 1.92 (s, 3H), 1.45 (s, 6H) ppm. ^13^C NMR (DMSO-*d*_6_, 125 MHz): *δ* 191.0, 187.4, 170.8, 164.3, 163.7, 155.7, 143.7, 137.2, 135.4, 134.1, 132.6, 132.5, 131.2, 131.0, 130.7, 129.7, 126.8, 125.9, 123.3, 119.1, 118.4, 116.8, 115.9, 53.3, 46.1, 45.6, 37.9, 28.6, 28.6, 21.0, 14.3, 13.1, 12.2 ppm.

#### 2.3.2. Synthesis of Probe **2**

Probe **2** was obtained from amide coupling of **4** with **7** by modifying the synthetic procedures for probe **1**. The resulting crude extract was purified by silica gel column chromatography (CH_2_Cl_2_:MeOH = 20:1), to give a yellow solid **2** in 29% yield. HR–ESI–MS *m*/*z* [M + H]^+^ calc. 570.2962, [M + H]^+^ obs. 570.2980 (mass error = 3.01 ppm). Purity determined by ^1^H NMR: 96%; ^1^H NMR (DMSO-*d*_6_, 500 MHz): *δ* 8.49–8.45 (m, 2H), 8.38–8.37 (d, *J* = 8.0 Hz, 1H), 7.82–7.79 (t, *J* = 7.8 Hz, 1H), 7.34–7.32 (d, *J* = 8.0 Hz, 1H), 4.04–4.00 (m, 2H), 3.81–3.71 (m, 4H), 3.21–3.11 (m, 4H), 3.06 (s, 1H), 2.05 (s, 3H), 1.88 (s, 3H), 1.87 (s, 3H), 1.63–1.57 (m, 2H), 1.40 (s, 6H) 1.37–1.32 (m, 2H), 0.94–0.91 (t, *J* = 7.3 Hz, 3H) ppm. ^13^C NMR (DMSO-*d*_6_, 125 MHz): *δ* 190.9, 187.4, 170.8, 163.9, 163.4, 155.7, 155.6, 143.7, 137.1, 135.4, 132.5, 131.1, 130.9, 129.5, 126.7, 125.9, 123.0, 116.5, 115.9, 60.2, 46.1, 45.6, 41.5, 37.9, 30.2, 28.6, 20.3, 14.2, 13.0, 12.2 ppm.

#### 2.3.3. Synthesis of Triphenylphosphonium-Naphthalimide-Piperazine **3**

4-Bromo-1,8-naphthalimide-triphenylphosphonium **5** (100 mg, 1.0 mmol) and piperazine (148 mg, 10 mmol) were dissolved in 2-methoxyethanol (20 mL), stirred and refluxed for 5 h under N_2_ atmosphere. The solvent was evaporated in vacuo, the residue was then purified using Waters Sep-Pak^®^ Vac 35 cc (10 g) C18 Cartridges. The residue was dissolved in H_2_O (10 mL) and loaded onto a C18 cartridge. The cartridge was then subject to an increasing gradient of CH_3_CN (0–50%) in H_2_O. The desired product **3** was eluted at about 20% of CH_3_CN. The collected fraction was dried *in vacuo* to give a yellow solid **3** (70 mg, 69%). HR–ESI–MS *m*/*z* [M]^+^ calc. 584.2461, [M]^+^ obs. 584.2408 (mass error = −9.57 ppm). ^1^H NMR (DMSO-*d*_6_, 500 MHz): *δ* 8.49–8.46 (m, 2H), 8.40–8.39 (d, *J* = 8.0 Hz, 1H), 7.91–7.98 (m, 3H), 7.83–7.75 (m, 13H), 7.36–7.34 (d, *J* = 8.0 Hz, 1H), 4.24–4.21 (t, *J* = 7.0 Hz, 2H), 3.78–3.72 (m, 2H), 3.22 (s, 4H), 3.12 (s, 4H), 1.98–1.96 (m, 3H) ppm. ^13^C NMR (DMSO-*d*_6_, 125 MHz): *δ* 162.4, 161.8, 159.4, 133.8, 132.4, 132.4, 129.1, 129.0, 127.8, 127.5, 127.4, 124.3, 123.8, 121.0, 117.4, 116.7, 113.7, 113.4, 52.4, 44.2, 43.2, 19.4, 17.3, 16.9 ppm.

#### 2.3.4. Synthesis of Butyl-Naphthalimide-Piperazine **4**

Butyl-naphthalimide-piperazine **4** was obtained from condensation of **6** with piperazine by modifying the synthetic procedures for **3**. A crude product was purified by a C18 cartridge (20% of CH_3_CN in H_2_O), giving a yellow and oily solid **4** in 79% yield. HR–ESI–MS *m*/*z* [M + H]^+^ calc. 338.1824, [M + H]^+^ obs. 338.1881 (mass error = 5.17 ppm). ^1^H NMR (DMSO-*d*_6_, 500 MHz): *δ* 8.44–8.41 (t, *J* = 7.8, 2H), 8.37–8.35 (d, *J* = 8.0 Hz, 1H), 7.79–7.76 (t, *J* = 7.8 Hz, 1H), 7.30–7.27 (d, *J* = 8.0 Hz, 1H), 4.02–4.00 (t, *J* = 7.5 Hz, 2H), 3.16–3.15 (m, 4H), 3.04–3.03 (m, 4H), 1.62–1.56 (m, 2H), 1.38–1.31 (m, 2H), 0.94–0.91 (t, *J* = 7.5 Hz, 3H) ppm. ^13^C NMR (DMSO-*d*_6_, 125 MHz): *δ* 164.0, 163.4, 156.6, 132.7, 131.0, 131.0, 129.6, 126.3, 125.7, 123.0, 115.8, 115.4, 54.2, 45.9, 39.6, 30.2, 20.3, 14.2 ppm.

## 3. Results and Discussion

### 3.1. Spectroscopic Analyses of Probes ***1*** and ***2*** to hNQO1 Activity

**Figure 1:**
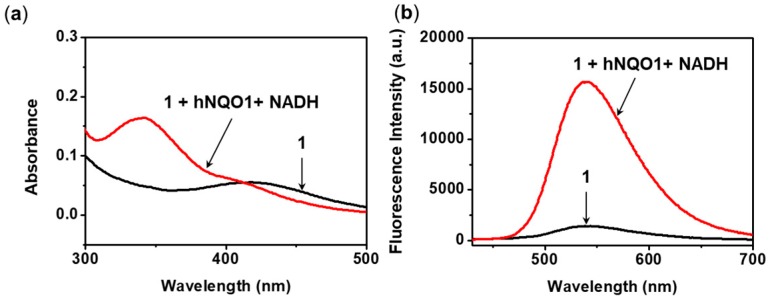
The absorption (**a**) and fluorescence (**b**) spectra of probe **1** (5 μM) in the absence (black) and presence (red) of hNQO1 (5 μg/mL) and NADH (100 μM). λ_ex_ = 410 nm.

The absorption and emission spectral changes of the probes towards hNQO1 activity were monitored in PBS solution (100 mM, pH = 7.4) containing 1% DMSO, 0.007% BSA and 0.1 M KCl at 37 °C. Probe **1** having a triphenylphosphonium salt showed a broad absorption at 435 nm, while upon the addition of hNQO1 and NADH, the absorption was slightly shifted to 400 nm (Figure 1a and Figure S1). On the other hand, probe **1** exhibited a very weak emission centered at 540 nm (Figure 1b). In the presence of hNQO1 and NADH, the emission intensity of probe **1** was significantly enhanced. It is proposed that the hNQO1-mediated cleavage of TLQ moiety results in the production of strongly fluorescent **3** as shown in Scheme 1. To verify the reaction mechanism, ESI-MS analyses were subsequently performed. We found that a mixture of probe **1** and hNQO1/NADH gave an apparent mass peak at 584.50 *m*/*z* corresponding to the expected product **3** (Figure S2). In addition, parallel analyses were carried out with probe **2**, which gave similar results to those of probe **1** (Figures S3 and S4).

**Figure 2:**
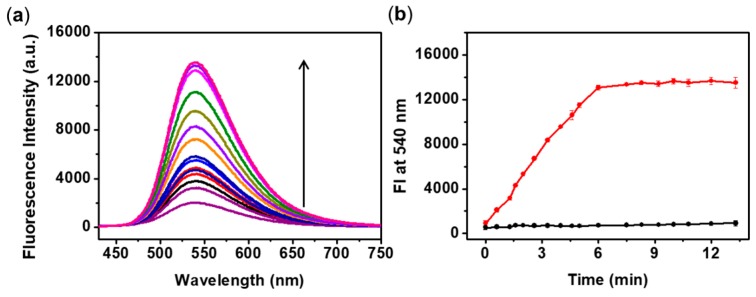
(**a**) Time-dependent fluorescence change of **1** (5 μM) in the presence of hNQO1 (5 μg/mL) and NADH (100 μM). (**b**) The fluorescence increase of probe **1** recorded at 540 nm with (red) and without (black) hNQO1 activity. Error bars indicate SD, n = 3. λ_ex_ = 410 nm.

The fluorescence increase of probe **1** towards hNQO1 activity was observed in a course of time. Upon mixing with hNQO1 and NADH, an instantaneous fluorescence enhancement of probe **1** was observed, and reached a plateau in 6 min (Figure 2) whereas in the absence of hNQO1 activity the probe **1** gave no fluorescence change. In the case of probe **2**, fluorescence increase was seen for 10 min and the increased intensity is lower than that of probe **1** (Figure S5). This indicates that the probe **1** responds to hNQO1 more rapidly than does probe **2**, presumably due to the triphenylphosphonium salt in probe **1** which promotes better solubility in aqueous media.

**Figure 3:**
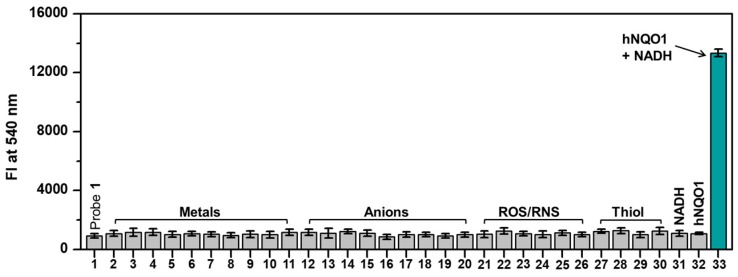
Fluorescence responses of probe **1** (5 μM) to metal ions (200 μM), anions (200 μM), reactive oxygen and nitrogen species (ROS and RNS) (200 μM), thiols (200 μM), NADH (100 μM), and hNQO1 (5 μg/mL). (1) Probe **1** only, (2) K^+^, (3) Na^+^, (4) Ca^2+^, (5) Mg^2+^, (6) Cu^+^, (7) Cu^2+^, (8) Co^2+^, (9) Zn^2+^, (10) Fe^2+^, (11) Fe^3+^, (12) Cl^−^, (13) F^−^, (14) I^−^, (15) ClO_4_^−^, (16) CN^−^, (17) H_2_PO_4_^−^, (18) HSO_4_^−^, (19) OAc^−^, (20) OH^−^, (21)·O_2_^−^, (22) H_2_O_2_, (23)·OH, (24) HOOtBu, (25)·OtBu, (26) NO, (27) glutathione, (28) cysteine, (29) homocysteine, (30) H_2_S, (31) NADH, (32) hNQO1, (33) hNQO1 and NADH. Error bars indicate SD, n = 3. λ_ex_ = 410 nm.

Fluorescence responses of probe **1** to other biologically potential interferants, including thiols, reactive oxygen and nitrogen species (ROS and RNS), anions, and metal ions were also investigated. Addition of hNQO1 activity revealed a significant fluorescence enhancement of probe **1** at 540 nm, whereas no such enhancement was observed in the case of other analytes (Figure 3 and cf. Figure S6 for probe **2**), implying that the probe **1** could be potentially used to detect hNQO1 activity in cancer cells excluding interference from other biologically abundant species.

**Figure 4:**
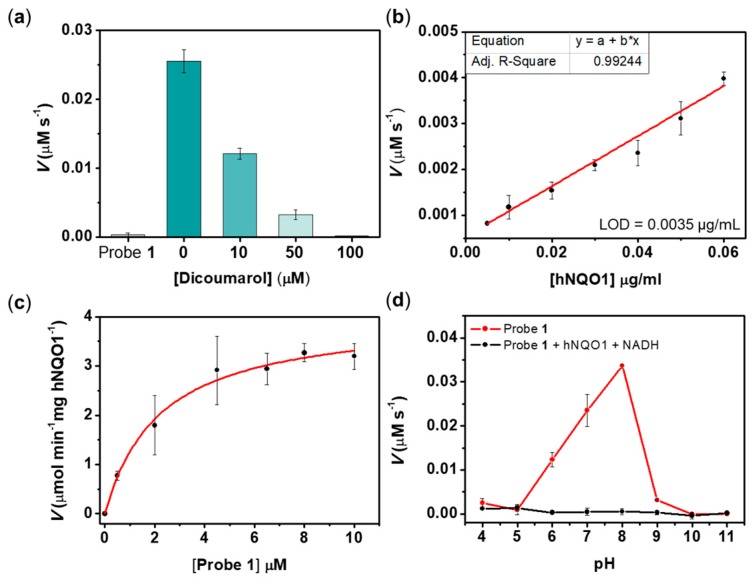
(**a**) Inhibitory assay of hNQO1-mediated fluorescence change of probe **1** using various concentrations of dicoumarol. All data were obtained by measuring the initial rates of fluorescence change of **1** (5 μM) at 540 nm toward hNQO1 (5 μg/mL) and NADH (100 μM) in the presence of various concentrations of dicoumarol (0, 10, 50, 100 μM). (**b**) The hNQO1-mediated fluorogenic reaction rates (*V*) of probe **1** at different concentrations of hNQO1. (**c**) The kinetics of hNQO1 using probe **1** based on Michaelis-Menten equation. (**d**) The pH effect on the enzymatic reaction of probe **1** (5 μM). All data were obtained by measuring the initial rates of fluorescence change at 540 nm in the absence (black) and presence (red) of hNQO1 (5 μg/mL) and NADH (100 μM). Error bars indicate SD, n = 3. λ_ex_ = 410 nm.

We then investigated a hNQO1-triggered fluorescence response of probe **1** in the presence of dicoumarol, hNQO1 inhibitor [17]. At different concentrations of dicoumarol (0, 10, 50, and 100 μM), the hNQO1-mediated fluorogenic reaction was found to be gradually inhibited (Figure 4a and cf. Figure S7 for probe **2**). In addition, the fluorescent response rate of probe **1** at various concentrations of hNQO1 was monitored. As seen in Figure 4b, probe **1** displayed a linear correlation between the rate and hNQO1 concentration (cf. Figure S8a for probe **2**), confirming that the fluorescence Off-On signal changed from probe **1** was driven by an hNQO1 activity as suggested in Scheme 1. In addition, limit of detection (LOD) and limit of quantification (LOQ) of probe **1** for NQO1 were calculated to be 0.0035 μg/mL and 0.0117 μg/mL, respectively [18]. Furthermore, the hNQO1-mediated reaction of probe **1** was analyzed based on the Michaelis-Menten equation (Figure 4c). The kinetic parameters were determined with Michaelis constant (*K*_m_) = 2.17 ± 0.46 μM, catalytic constant (*k*_cat_) = 2.07 ± 0.13 s^−1^, maximum velocity (*V*_max_) = 4.01 ± 0.24 μmol min^−1^ mg hNQO1^−1^, and specificity constant (*k*_cat_/*K*_m_) = (9.54 ± 0.20) × 10^5^ M^−1^ s^−1^ (cf. Figure S8b for probe **2**).

The pH effect on the hNQO1-mediated fluorogenic reaction of probe **1** was also investigated. Probe **1** showed a noticeable fluorescence increase towards hNQO1 in pH range of 5–9 (Figure 4d and cf. Figure S9 for probe **2**). However, without hNQO1, probe **1** does not show any fluorescence change in those pH range. This also implies that probe **1** could be used as a hNQO1-responsive fluorescent turn-on probe in most of the physiological cellular pH.

Prior to the application of our system for imaging of hNQO1 in cancer cells, we compared detection capabilities (detection time, emission wavelength range, and specificity constant) of the system with other fluorescent probes previously reported (Table 1). It is known that specificity constant determines enzymatic reaction rate and affinity with the enzyme. Notably, probe **1** provided an enhanced specificity constant, rapid detection, and an emission at a longer wavelength (>500 nm) compared to other reported probes. In addition, a specificity constant of probe **1** is superior to the **2**. As the results, we could propose that probe **1** is highly efficient for hNQO1 detection, which might be due to an improved solubility in the biological media.

### 3.2. Detection Capabilities of Probe ***1*** to Cellular hNQO1 in Cancer Cells

**Figure 5:**
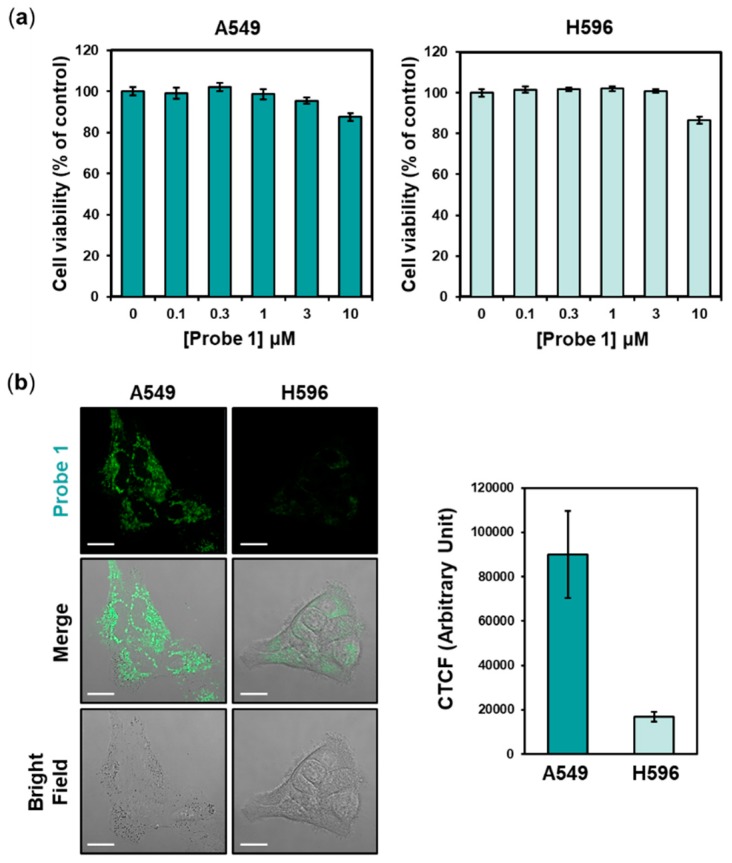
(**a**) Cell viability of probe **1** in A549 (hNQO1-positive) and H596 (hNQO1-negative) cells. Cells were incubated with 0, 0.1, 0.3, 1.0, 3.0, and 10 μM of probe **1** for 24 h. Independent experiments are performed in triplicate. (**b**) Confocal images of hNQO1-positive A549 and hNQO1-negative H596 cells treated with probe **1** (5 μM) for 1 h at 37 °C. Bar graph represents the corrected total cell fluorescence (CTCF) of confocal images of probe **1**. λ_ex_ = 405 nm; λ_em_ = 430–540 nm. Scale bar = 20 μm.

Encouraged by the excellent optical properties of probe **1**, we evaluated the detection capabilities of **1** for monitoring hNQO1 activity in the live cells. Prior to cellular imaging, cytotoxicity of **1** was estimated in two different lung cancer cell lines, hNQO1-positive A549 and hNQO1-negative H596 cell lines (Figure 5a). The probe **1** showed the negligible cytotoxic effects in A549 and H596 cells, even after 24 h incubation (cf. Figure S10 for probe **2**). In addition, the cell viability was tested at different incubation time of the probes (Figure S11). Moreover, it was checked that the probes does not induce apoptosis. For the optimization of cell imaging, the cells were treated with different concentration of the probes, and the fluorescence images were monitored in the course of incubation time (Figures S12a,b,d,e and S13). We also performed the quantitative analysis of the probes’ cell uptake via flow cytometry (Figure S12c,f). Probe **1** showed a time-dependent increase in the number of fluorescent cells. However, the time-dependent increase was not seen in the case of probe **2** probably due to other biological independent factors such as probes’ different cell uptake pathway, enzyme efficiency, etc. It is known that various uptake pathways are involved in an internalization of molecules into cells. It was suggested that butyl-naphthalimide-disulfide seemed to be uptaken into the cells by a caveolae pathway [19], where the chemical structure is similar to probes **1** and **2**. Although it was expected that the probes can be uptaken into the cells with the similar pathway, further experiments are necessary to understand the exact uptake mechanism. We checked that internalization of the probes into the cells is independent of organic anion transporting polypeptides (OATPs) [20], confirmed by OATPs inhibition test in A549 cells. However, further study is required to know the exact uptake mechanism. Confocal microscopy was then carried out using probe **1**, to demonstrate if the probe give a fluorescence response to hNQO1 in living cells. We discovered that the probe **1**-treated hNQO1-positive A549 cells showed a significant green fluorescence, whereas a relatively weak fluorescence was seen in the hNQO1-negative H596 cells (Figure 5b and cf. Figure S14 for probe **2**).

**Figure 6:**
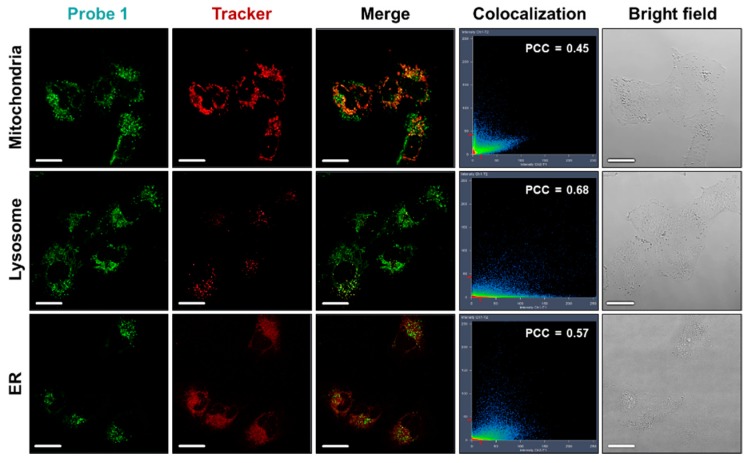
Colocalization images of probe **1** and suborganelles in A549 cells. The cells were incubated with probe **1** (5 μM) for 1 h at 37 °C. The organelles, such as mitochondria, lysosomes, and endoplasmic reticulum (ER), are stained using organelle-selective trackers. The colocalization images were analyzed by the ZEN software (blue edition version) and PCC values indicated Pearson’s correlation coefficient. Scale bar = 20 μM.

We then investigated the cellular location of probe **1** in hNQO1-positive A549 cells using organelle-selective staining dyes, such as MitoView633 for mitochondria, LysoView633 for lysosomes, and ER-Tracker Red for endoplasmic reticulum (ER). The cells were treated with probe **1** and organelle staining dyes (Figure 6). Pearson’s correlation coefficient (PCC) of probe 1 for the mitochondria, lysosomes and ER staining dyes turned out to be 0.45, 0.68, and 0.57, respectively (cf. Figure S15 for probe **2**). This indicated that probe **1** located in the mitochondria, lysosomes and ER, and thus probe **1** might be used to detect the hNQO1 activity in wide range of compartments of living cells.

**Figure 7:**
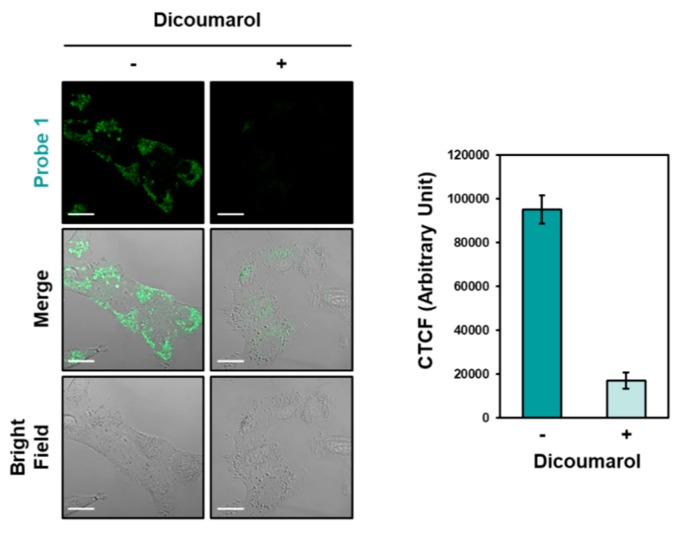
Confocal images of hNQO1-positive A549 cells after pretreatment with 400 μM of dicoumarol for 6 h. The cells were incubated with probe **1** (5 μM) for 1 h at 37 °C. Bar graph represents the corrected total cell fluorescence (CTCF) of confocal images of probe **1**. λ_ex_ = 405 nm; λ_em_ = 430–540 nm. Scale bar = 20 μm.

Moreover, we checked if the fluorescence images obtained from the probe **1** treated A549 cells are caused by hNQO1. A549 cells were incubated with probe **1** in pretreatment of hNQO1 inhibitor, dicoumarol which competes with NADH, thereby inhibiting hNQO1 activity [17]. As expected, in the presence of dicoumarol, probe **1** showed significantly decreased fluorescence intensity in A549 cells (Figure 7 and cf. Figure S16 for probe **2**). These results firmly confirmed that probe **1** could selectively respond to the hNQO1 activity in living cells.

Therefore, we believed that this system can be employed for detecting and imaging of hNQO1 activity in biological milieu such as living cells and it can be a promising analytical tool for cancer diagnosis related with hNQO1.

## 4. Conclusions

We developed cancer-specific hNQO1 responsive naphthalimides (probes **1** and **2**), which can provide a fluorescent Off-On signal at relatively long wavelength range of 500–650 nm through a hNQO1-mediated TLQ cleavage. In addition, probe **1**, a TLQ linked naphthalimide containing a triphenylphosphonium salt, showed a good solubility leading to a high enzyme efficiency and rapid detection under the physiological conditions. Moreover, probe **1** was found to be quite biocompatible and it readily provided a strong fluorescence image without interfering by autofluorescence in hNQO1-overexpressed cancer cells (A549). The detection capability of probe **1** to hNQO1 was definitely proven by using the hNQO1-negative H596 and hNQO1 inhibitor-pretreated A549 cells. We proposed that this system might be potentially useful for the diagnostic and therapeutic developments of hNQO1-related cancers.

## Figures and Tables

**Scheme 2:**
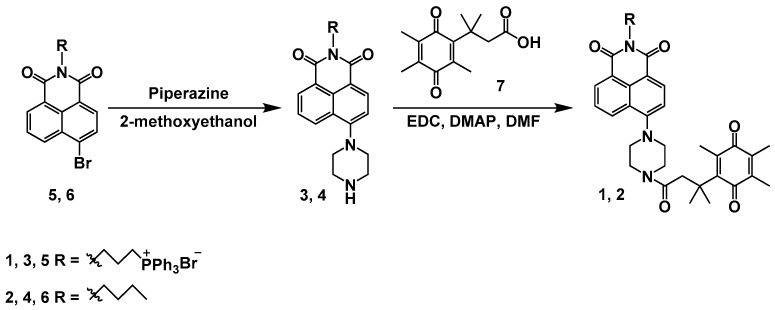
Synthetic pathway for probes **1** and **2**. Conditions: reflux for 4 h at 140 °C, yields: **3** (69%) and **4** (79%); room temperature for 14 h, yields: **1** (32%) and **2** (29%).

**Table 1:** The hNQO1 detection abilities of probes in this work and previously reported fluorescent probes.

Probe	Detection Time	Emission	Specificity Constant (*k*_cat_/*K_m_*)	Ref.
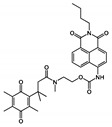	37 min	470 nm	(4.94 ± 0.33) × 10^3^ M^−1^ s^−1^	[8]
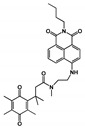	N/A	527 nm	(3.2 ± 0.6) × 10^4^ M^−1^ s^−1^	[9]
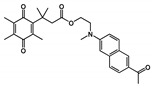	15 min	520 nm	2.78 × 10^5^ M^−1^ s^−1^	[10]
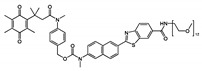	12 min	450 nm & 540 nm	4.61 × 10^3^ M^−1^ s^−1^	[11]
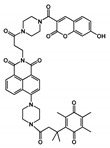	60 min	460 nm & 540 nm	(7.66 ± 0.02) × 10^4^ M^−1^ s^−1^	[12]
Probe **2**	10 min	540 nm	(4.03 ± 0.14) × 10^5^ M^−1^ s^−1^	This work
Probe **1**	6 min	540 nm	(9.54 ± 0.20) × 10^5^ M^−1^ s^−1^	This work

## Supplementary Materials

The following are available online at https://www.mdpi.com/1424-8220/20/1/53/s1.
