# Supplementary material for: Cancer-Specific hNQO1-Responsive Biocompatible Naphthalimides Providing a Rapid Fluorescent Turn-On with an Enhanced Enzyme Affinity
Source: Sensors (Basel). 2019 Dec 20;20(1):53. doi: 10.3390/s20010053 (PMC6982707; doi:10.3390/s20010053)
Supplement: Supplementary file 1 [file sensors-20-00053-s001.pdf]

Supporting Information

# Cancer-specific hNQO1-responsive biocompatible naphthalimides providing a rapid fluorescent turn-on with an enhanced enzyme affinity

**Sun Young Park[a,†], Eugeine Jung[b,†], Jong Seung Kim[c,*], Sung-Gil Chi[b,*] and Min Hee Lee[a,*]**

[a]*Department of Chemistry, Sookmyung Women's University, Seoul 04310, Korea*

[b]*Department of Life Sciences, Korea University, Seoul 02841, Korea*

[c]*Department of Chemistry, Korea University, Seoul 02841, Korea*

[†] These authors contributed equally to this work.

**Contents**

1. **UV/Vis absorption and fluorescence spectroscopic methods**
2. **Enzyme titration**
3. **Enzyme inhibition**
4. **Enzyme kinetics**
5. **Cell viability assay**
6. **Flow cytometry analysis**
7. **Colocalization imaging**
8. **Additional data**
9. **$^{1}$H and $^{13}$C NMR and HR-ESI-MS analyses**

## 1. UV/Vis absorption and fluorescence spectroscopic methods

Stock solutions of synthetic probes were prepared in DMSO. Stock solutions of metal chloride salts and thiols were prepared in deionized water. Stock solutions of TBA salts anions and $[Cu(CH_3CN)_4]PF_6$ for $Cu^+$ ion were prepared in $CH_3CN$. Stock solutions of reactive oxygen species (ROS) were prepared by using literature procedures.[1] All spectra were recorded in PBS/DMSO solution (v/v = 99:1, 100 mM, pH = 7.4) containing BSA (0.007%) and KCl (0.1 M) at 37 °C. Excitation was carried out at 410 nm with all excitation slit widths 5 nm.

## 2. Enzyme titration

Stock solutions of hNQO1 and NADH were prepared in deionized water. Time dependent of fluorescence response of **1** (5 μM) or **2** (5 μM) to various concentrations of hNQO1 in the presence of NADH (100 μM) were determined at 540 nm, with a time interval of 0.1 s for 5 min. All measurements were recorded in PBS/DMSO solution (v/v = 99:1, 100 mM, pH = 7.4) containing BSA (0.007%) and KCl (0.1 M) in 37 °C. $\lambda_{ex}$ = 410 nm. The fluorescence units were converted to concentration by relating the signal increase to a fluorescence signal derived from a known concentration of compound **3** or **4**. From this, velocity (μM $s^{-1}$) was plotted as a function of [hNQO1] (μM/mL).

## 3. Enzyme inhibition

Stock solution of dicoumarol (3,3'-methylene-bis(4-hydroxycoumarin)) was prepared in DMSO. Time dependent fluorescence response of **1** (5 μM) or **2** (5 μM) to hNQO1 (5 μg/mL) and NADH (100 μM) in the presence of various concentrations of dicoumarol (0, 10, 50 and 100 μM) were determined at 540 nm, with a time interval of 0.1 s for 5 min. All measurements were recorded in PBS/DMSO solution (v/v = 96:4, 100 mM, pH = 7.4) containing BSA (0.007%) and KCl (0.1 M). $\lambda_{ex}$ = 410 nm.

## 4. Enzyme kinetics

Time dependences of fluorescence response of various concentrations of **1** (0-0.06 μM) or **2** (0-0.5 μM) to hNQO1 (0.5 μg/mL) in the presence of NADH (100 μM) were determined at 560 nm, with a time interval of 0.1 s for 5 min. All measurements were recorded in PBS/DMSO solution (v/v = 99:1, 100 mM, pH = 7.4) containing BSA (0.007%) and KCl (0.1 M). $\lambda_{ex}$ = 410 nm. The fluorescence units were converted to concentration by relating the signal increase to a fluorescence signal derived from a known concentration of compounds **3** and **4**. Velocity (μmol $min^{-1}$ $mghNQO1^{-1}$) was plotted as a function of [probes] (μM) to obtain $K_m$ and $V_{max}$ values from nonlinear least-squared analysis for Michaelis-Menten kinetics (OriginPro 8.0). From this, $k_{cat}$ and $k_{cat}/K_m$ values were calculated from these values.

---

1. R. D. Rohde, H. D. Agnew, W.-S. Yeo, R. C. Bailey, and J. R. Heath, *J. Am. Chem. Soc.,* 2006, **128**, 9518.

## 5. Cell viability assay

A549 cells ($2\times10^4$/well) and H596 cells ($0.9\times10^4$/well) were seeded on 96 well microplate (SPL Life Science) and incubated for 1 h, 24 h and 36 h, respectively. After the cells were stabilized, 1% DMSO was used as a control and probes **1** or **2** was treated for indicated time points. To analyze the viability of cells in the presence and absence of probes **1** and **2**, the CytoTox96® Non-Radioactive Cytotoxicity Assay Kit (Promega, Madison, WI, USA) was used, following the manufacturer's protocols. Power Wave XS Microplate Reader (Biotek Instruments Inc., Winooski, VT, USA) was used to measure the fluorescence level. The wavelength was set at 492 nm. Cell viability assays were performed in triplicates, and the viability was expressed as a percentage (%) of measured absorbance, relative to the control cells.

## 6. Flow cytometry analysis

Flow cytometry was used for quantitative analysis of cellular internalization of probes **1** and **2**. A549 cells were seeded ($1\times10^5$/mL, total volume 4 mL) in 60 mm culture dish (SPL Life Science, Seoul, Republic of Korea) and incubated for 24 h. Cells were treated with 5 µM of probes **1** or **2** and incubated for 10 min, 30 min, and 1 h, respectively. Non-treated cells were used as the control for experiments. All cells were detached from the dish using 0.05 % trypsin-EDTA solution (Welgene, Daegu, Republic of Korea) and washed with PBS for two times before flow cytometry analysis. Fluorescence-activated cell sorting flow cytometry system (FACS Calibur BD Flow Cytometer, BD Biosciences, California, USA) was used for cells analysis.

## 7. Colocalization imaging

In order to verify the hNQO1 visualization abilities of probes **1** and **2**, the cellular imaging and colocalization images were taken on hNQO1-positive A549 cells. The cells were seeded ($2.5\times10^4$/ml, total volume 2 ml) in glass-bottomed confocal dishes (SPL Life Science) and incubated in the same conditions as mentioned above. The cells were treated with probe **1** (5 µM) or **2** (5 µM) for 1 h and stained with suborganelles trackers. MitoView633 (Biotium Inc., Fremont, CA, USA) was used for mitochondria staining, LysoView633 (Biotium Inc., Fremont, CA, USA) for lysosome and ER-tracker Red (Invitrogen Inc., Carlsbad, CA, USA) for endoplasmic reticulum (ER). The cells were incubated with 500 nM of MitoView633 or LysoView633 for 30 min, or with 2 µM ER-tracker Red for 1 h. The fluorescence channel was excited at 555 nm and the emission was recorded at 600-700 nm with a band-pass filter. The dishes were washed by PBS three times to remove free dyes and the fluorescence was imaged. The colocalization images were analyzed by the ZEN software (blue sedition version). And the level of CTCF (Corrected total cell fluorescence) was calculated using the following formula, with the data from confocal microscopy images, using ImageJ software: CTCF = Integrated Density – (Area of selected cell × Mean fluorescence of background readings). Magnification of each image is 40×.

## 8. Additional Data

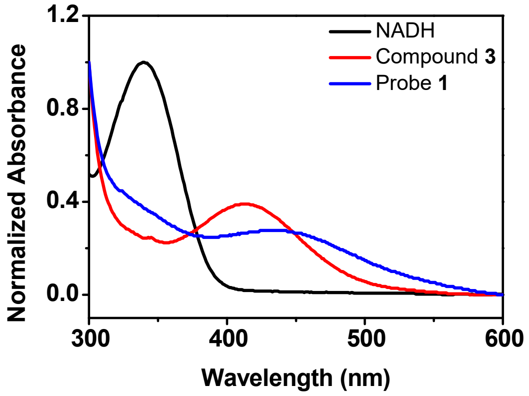


**Figure S1.** The normalized absorption spectra of probe **1**, compound **3** and NADH.

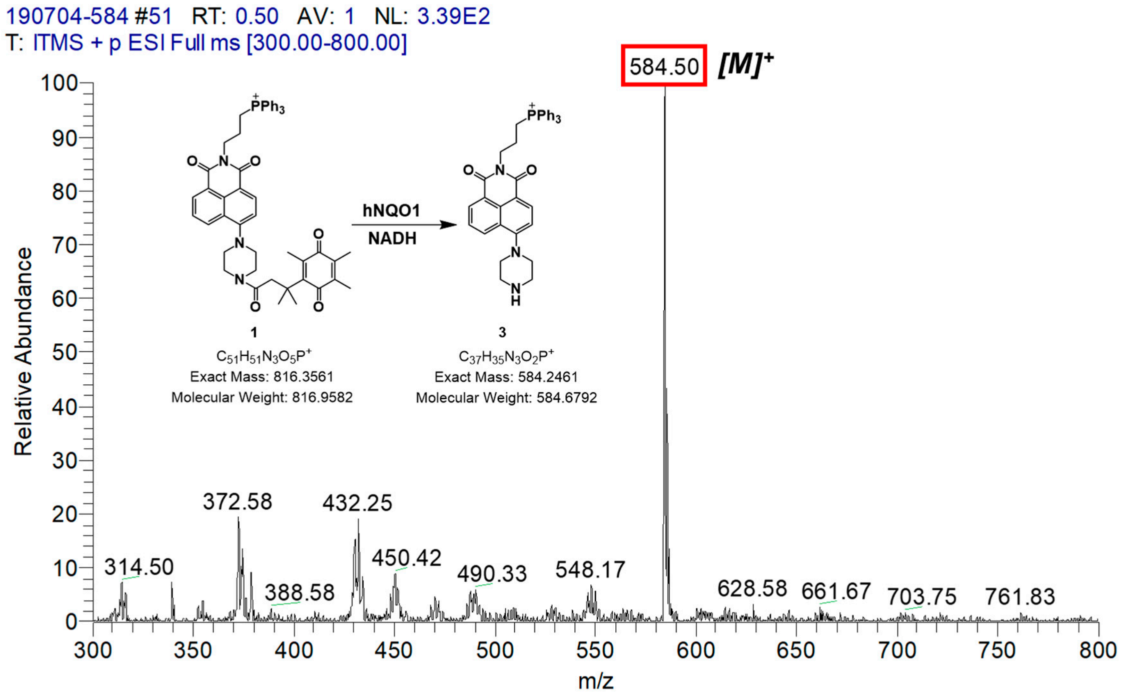


**Figure S2.** ESI-MS spectrum of **1** in the presence of hNQO1 and NADH.

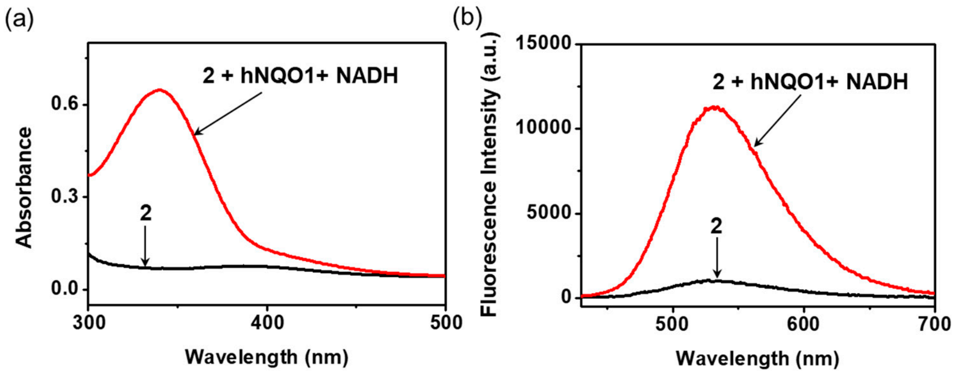


**Figure S3.** (a) Absorption and (b) emission spectra of **2** (5 μM) in absence and presence of hNQO1 (5 μg/mL) and NADH (100 μM). All data were obtained in PBS/DMSO solution (v/v = 99:1, 100 mM, pH = 7.4) containing BSA (0.007%) and KCl (0.1 M) at 37℃. $\lambda_{ex}$ = 410 nm.

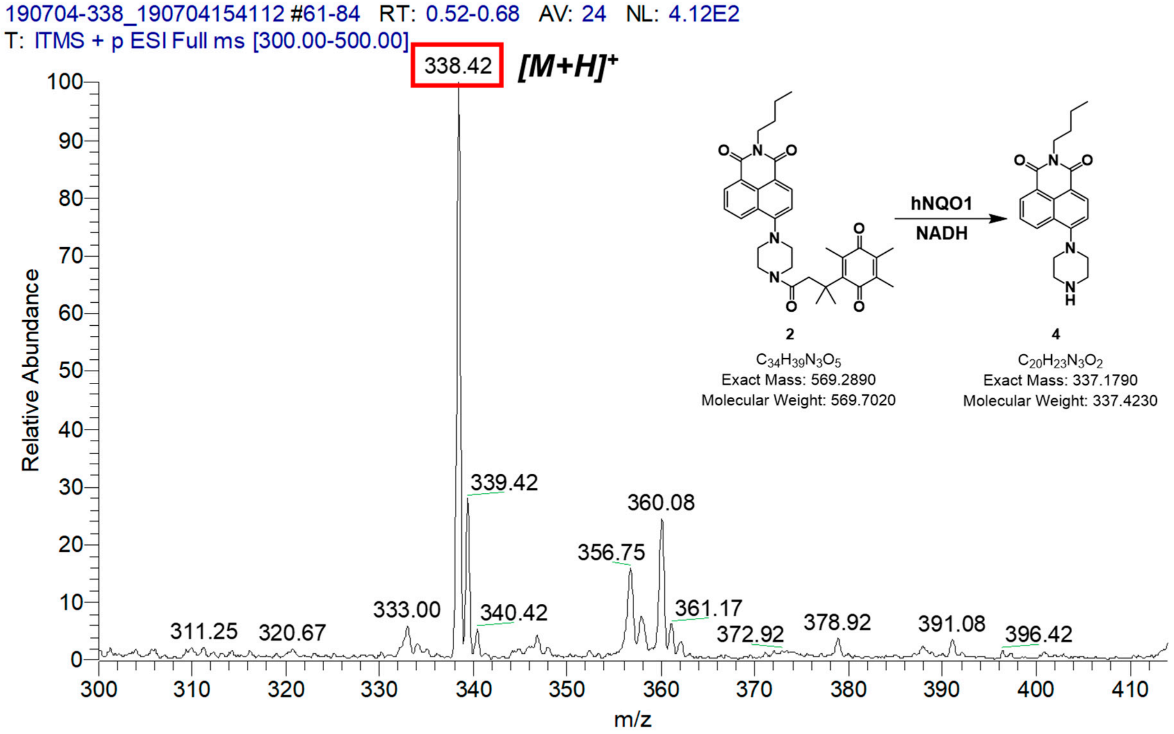


**Figure S4.** ESI-MS spectrum of **2** in the presence of hNQO1 and NADH.

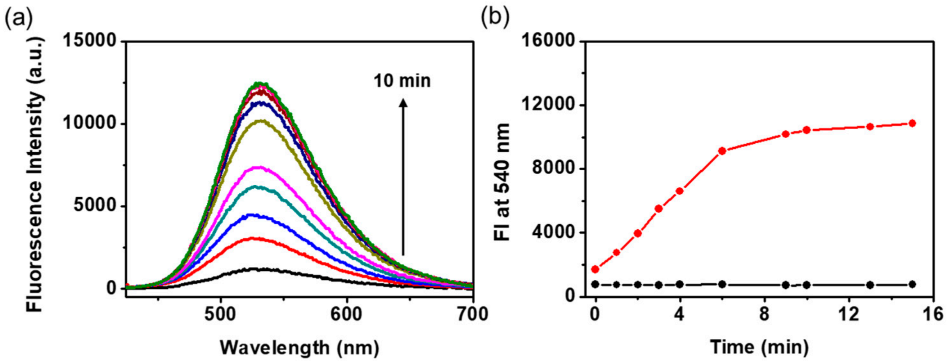


**Figure S5.** (a) Time-dependent fluorescence change (0 - 10 min) of **2** (5 μM) in the presence of hNQO1 (5 μg/mL) and NADH (100 μM). (b) Fluorescence intensity at 540 nm of probe **2** in the presence (red) and absence (black) of hNQO1 and NADH. All spectra were recorded in PBS/DMSO solution (v/v = 99:1, 100 mM, pH = 7.4) containing BSA (0.007%) and KCl (0.1 M) at 37 °C. $\lambda_{ex}$ = 410 nm.

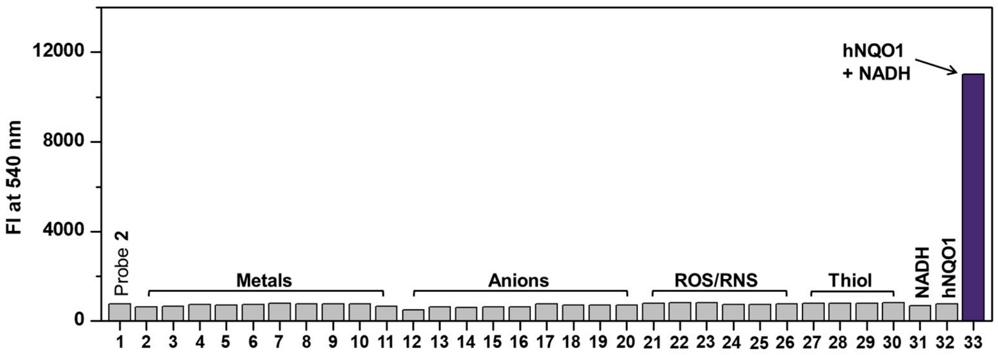


**Figure S6.** Fluorescence intensity at 540 nm of **2** (5 μM) in the presence of various biological relevant metals (200 μM), anions (200 μM), reactive oxygen/nitrogen species (ROS/RNS) (200 μM), thiols (200 μM), NADH (100 μM), and hNQO1 (5 μg/mL). (1) Probe **2** only, (2) $K^+$, (3) $Na^+$, (4) $Ca^{2+}$, (5) $Mg^{2+}$, (6) $Cu^+$, (7) $Cu^{2+}$, (8) $Co^{2+}$, (9) $Zn^{2+}$, (10) $Fe^{2+}$, (11) $Fe^{3+}$, (12) $Cl^-$, (13) $F^-$, (14) $I^-$, (15) $ClO_4^-$, (16) $CN^-$, (17) $H_2PO_4^-$, (18) $HSO_4^-$, (19) $OAc^-$, (20) $OH^-$, (21) $\cdot O_2^-$, (22) $H_2O_2$, (23) ·OH, (24) HOOtBu, (25) ·OtBu, (26) NO, (27) glutathione, (28) cysteine, (29) homocysteine, (30) $H_2S$, (31) NADH, (32) hNQO1, (33) hNQO1 and NADH. All data were collected in PBS/DMSO solution (v/v = 99:1, 100 mM, pH = 7.4) containing BSA (0.007%) and KCl (0.1 M) at 37 °C. $\lambda_{ex}$ = 410 nm.

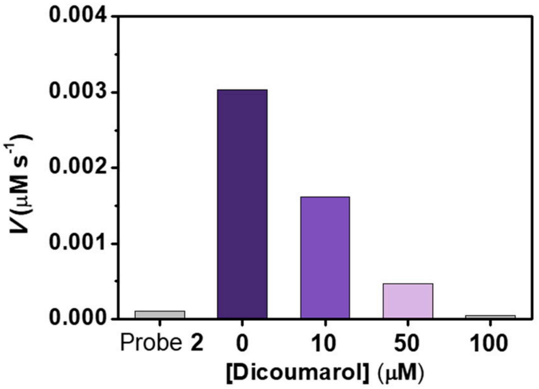


**Figure S7.** Inhibitory assay of hNQO1 activity-based fluorescence change of **2** using various concentrations of dicoumarol. All data were obtained by measuring the initial rates of fluorescence change of **2** (5 μM) at 540 nm toward hNQO1 (5 μg/ml) and NADH (100 μM) in the presence of various concentrations of dicoumarol (0, 10, 50, 100 μM). All data were obtained in PBS/DMSO solution (v/v = 96:4, 100 mM, pH = 7.4) containing BSA (0.007%) and KCl (0.1 M). $\lambda_{ex}$ = 410 nm.

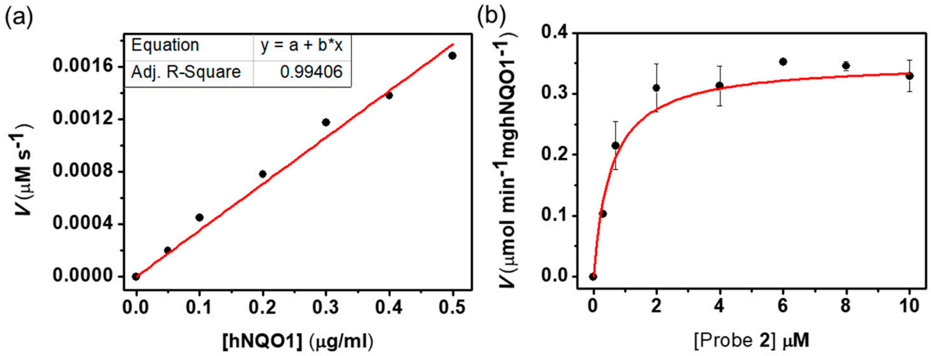


**Figure S8.** (a) Enzyme titration of hNQO1 (0-0.5 μg/mL) with probe **2**. (b) Enzyme kinetics of hNQO1 (0.5 μg/mL) with probe **2** (mean ± sd, n = 3). The kinetic parameters were estimated to be $K_m$ = 0.48 ± 0.13 μM, $k_{cat}$ = 0.20 ± 0.009 $s^{-1}$, $V_{max}$ = 0.38 ± 0.02 μmol $min^{-1}$ mg $hNQO1^{-1}$, and $k_{cat}/K_m$ = (4.03 ± 0.14) x $10^5$ $M^{-1}$ $s^{-1}$. All spectra were recorded in PBS/DMSO solution (v/v = 99:1, 100 mM, pH = 7.4) containing BSA (0.007%) and KCl (0.1 M) at 37 °C. $\lambda_{ex}$ = 410 nm.

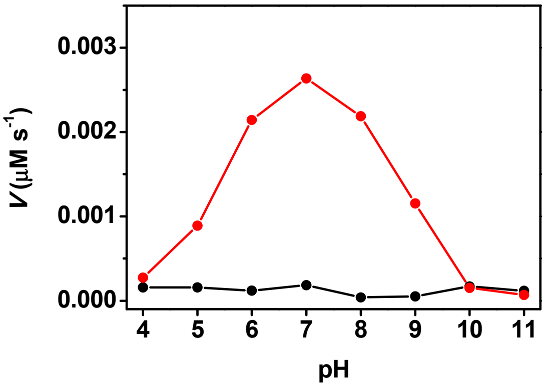


**Figure S9.** The pH effect on the enzymatic reactions of **2** (5 μM, respectively). All data were obtained by measuring the initial rates of fluorescence change at 540 nm in the absence (black) and presence (red) of hNQO1 (5 μg/ml) and NADH (100 μM) in different pH buffer solutions. $\lambda_{ex}$ = 410 nm.

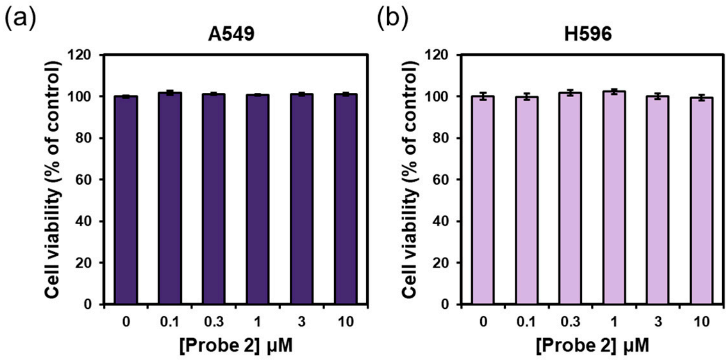


**Figure S10.** Cell viability of probe **2** in (a) A549 (hNQO1-positive), (b) H596 (hNQO1-negative) cells. Cells were incubated with 0, 0.1, 0.3, 1.0, 3.0 and 10 μM of probe **2** for 24 h. Independent experiments are performed in triplicate.

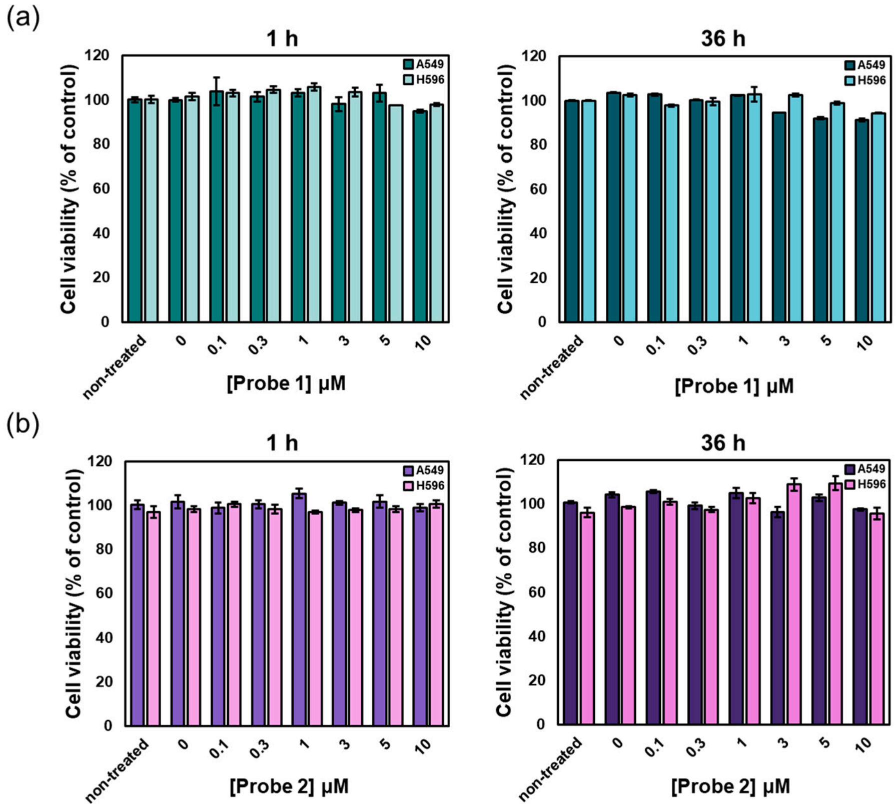


**Figure S11.** Cell viability of probes (a) **1** and (b) **2** for 1 h and 36 h incubations in A549 cells (hNQO1-positive) and H596 (hNQO1-negative) cells. Cells were incubated with different concentration of the probes **1** and **2** at media containing 1% of DMSO, except for the non-treated.

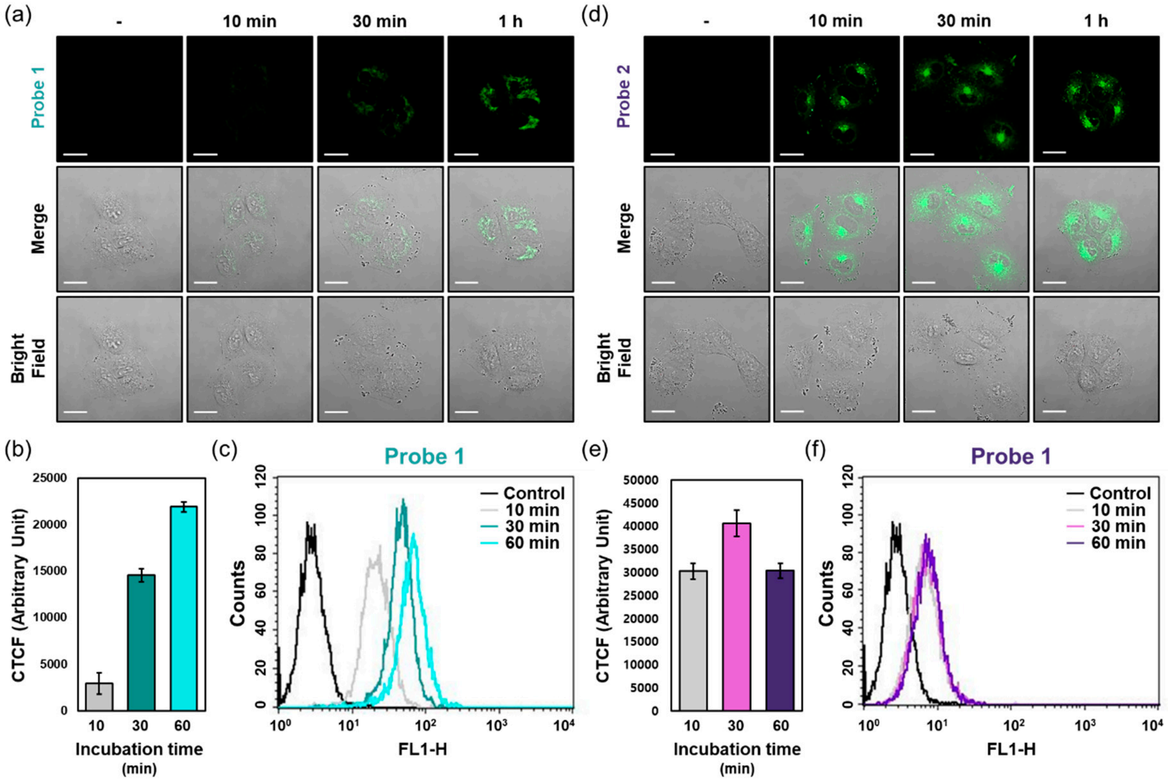


**Figure S12.** Time-dependent confocal images of hNQO1-positive A549 cells after treated with probes (a) **1** and (d) **2** (5 μM) at 37 °C. $\lambda_{ex}$ = 405 nm; $\lambda_{em}$ = 430–540 nm. Scale bar = 20 μm. Bar graphs (b) and (e) represent the corrected total cell fluorescence (CTCF) of confocal images of probes **1** and **2**. Flow cytometry analysis with probes (c) **1** and (f) **2** in A549 cells. The cells were treated with probes (5 μM) at 37 °C for each incubation time. Black color indicates the non-treated cells (control) showing a background fluorescence.

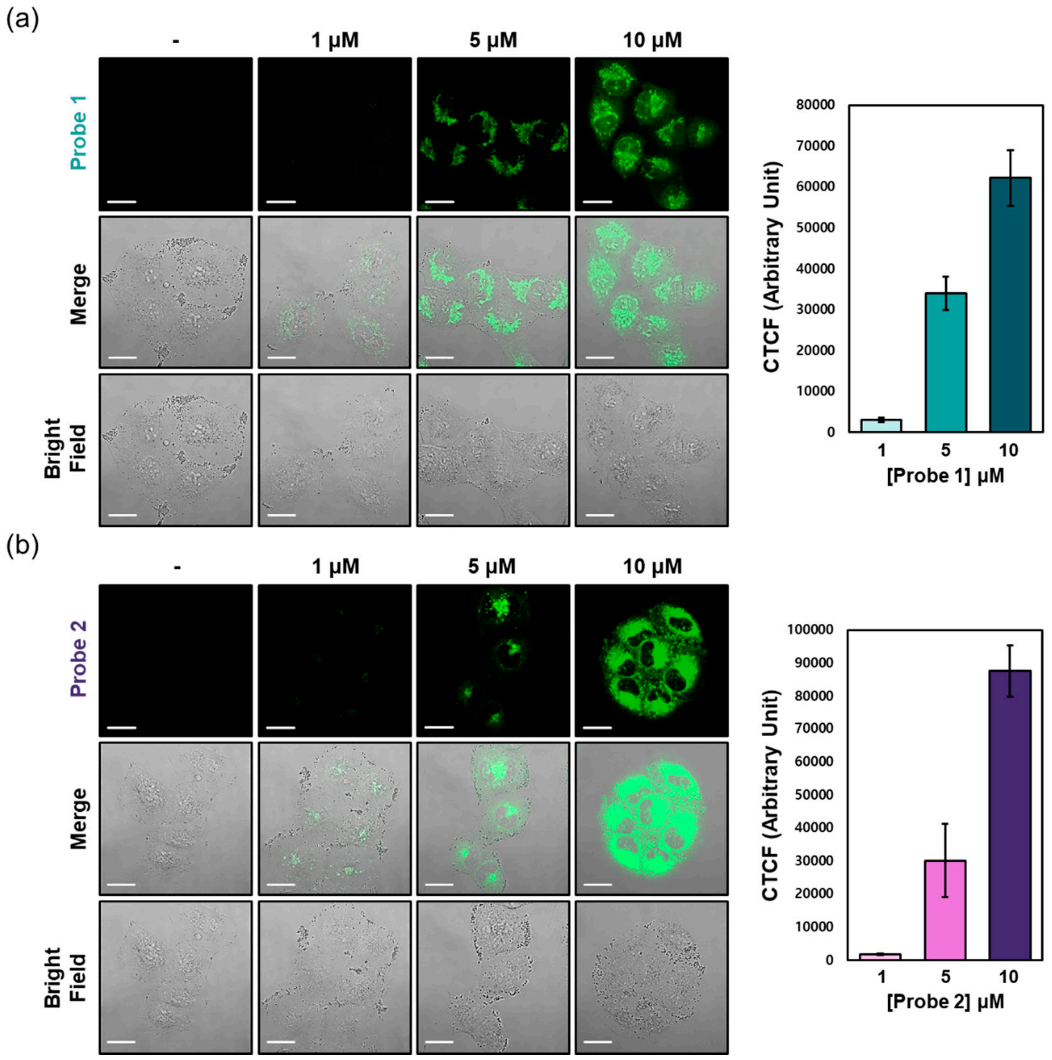


**Figure S13.** Confocal images of hNQO1-positive A549 cells treated with different concentration (1, 5 and 10 μM) of probes (a) **1** and (b) **2** for 1 h at 37 °C. Bar graphs represent the corrected total cell fluorescence (CTCF) of confocal images of each probe. $\lambda_{ex}$ = 405 nm; $\lambda_{em}$ = 430–540 nm. Scale bar = 20 μm.

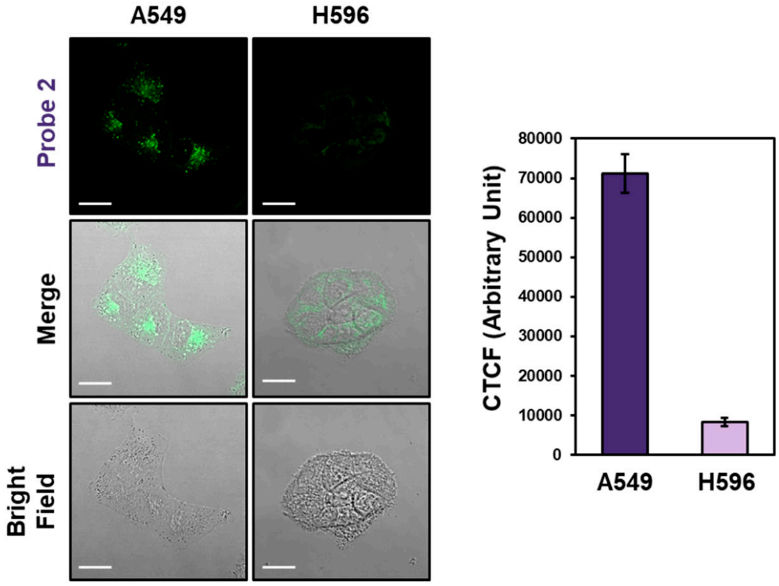


**Figure S14.** Confocal images of hNQO1-positive A549 and hNQO1-negative H596 cells treated with probe **2** (5 μM) for 1 h at 37 °C. Bar graph represents the corrected total cell fluorescence (CTCF) of confocal images of probe **2**. $\lambda_{ex}$ = 405 nm; $\lambda_{em}$ = 430–540 nm. Scale bar = 20 μm.

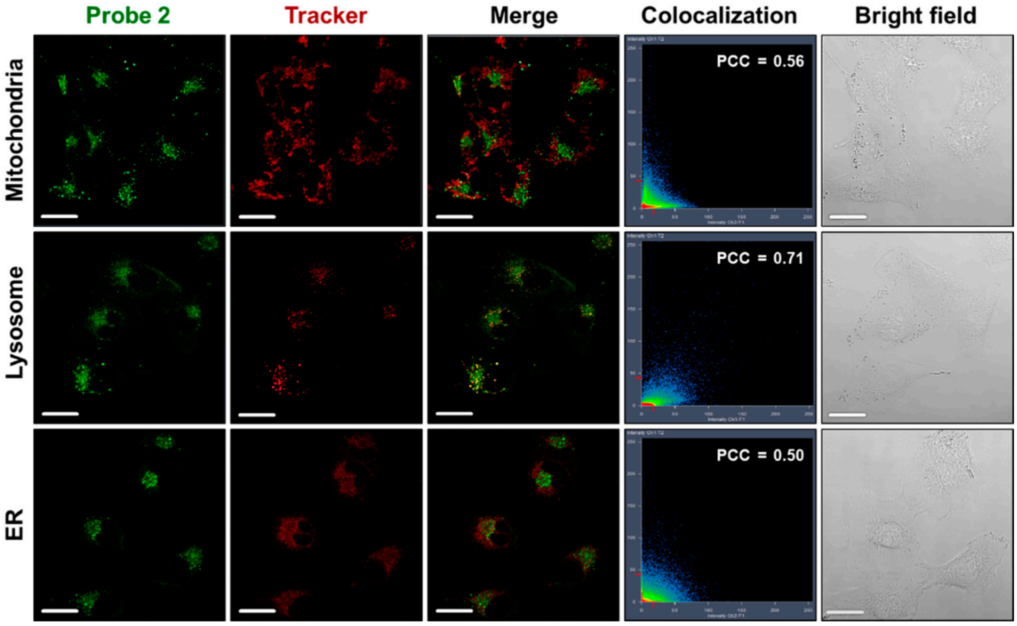


**Figure S15.** Colocalization images of probe **2** and suborganelles in A549 cells. The cells were incubated with probe **2** (5 μM) for 1 h at 37°C. The organelles, such as mitochondria, lysosomes and endoplasmic reticulum (ER), are stained using organelle-selective trackers. The colocalization images were analyzed by the ZEN software (blue edition version) and PCC values indicated Pearson's correlation coefficient. Scale bar = 20 μm.

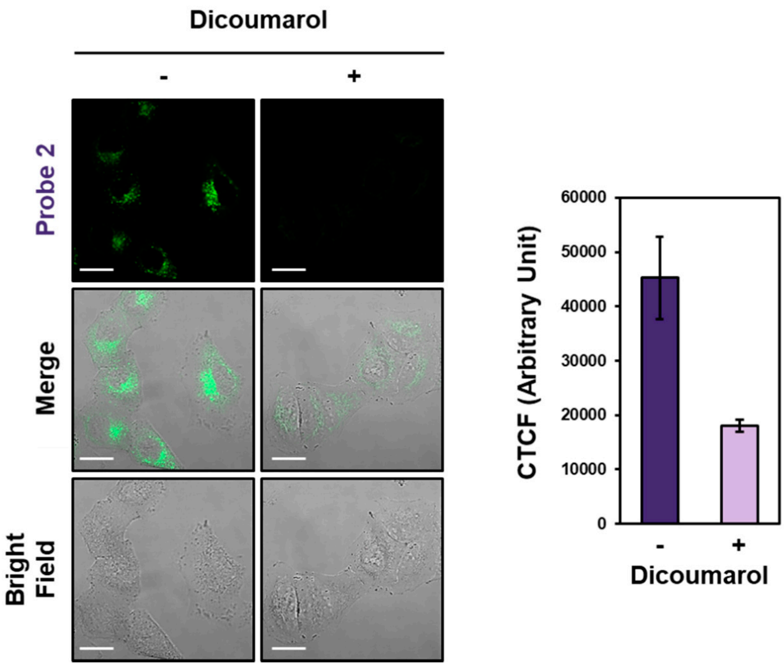


**Figure S16.** Confocal images of hNQO1-positive A549 cells after pretreatment with 400 μM of dicoumarol for 6 h. The cells were incubated with probe **2** (5 μM) for 1 h at 37 °C. $\lambda_{ex}$ = 405 nm; $\lambda_{em}$ = 430–540 nm. Scale bar = 20 μm. Bar graph represents the corrected total cell fluorescence (CTCF) of confocal images.

## 9. $^{1}H$ and $^{13}C$ NMR and HR-ESI-MS Analyses

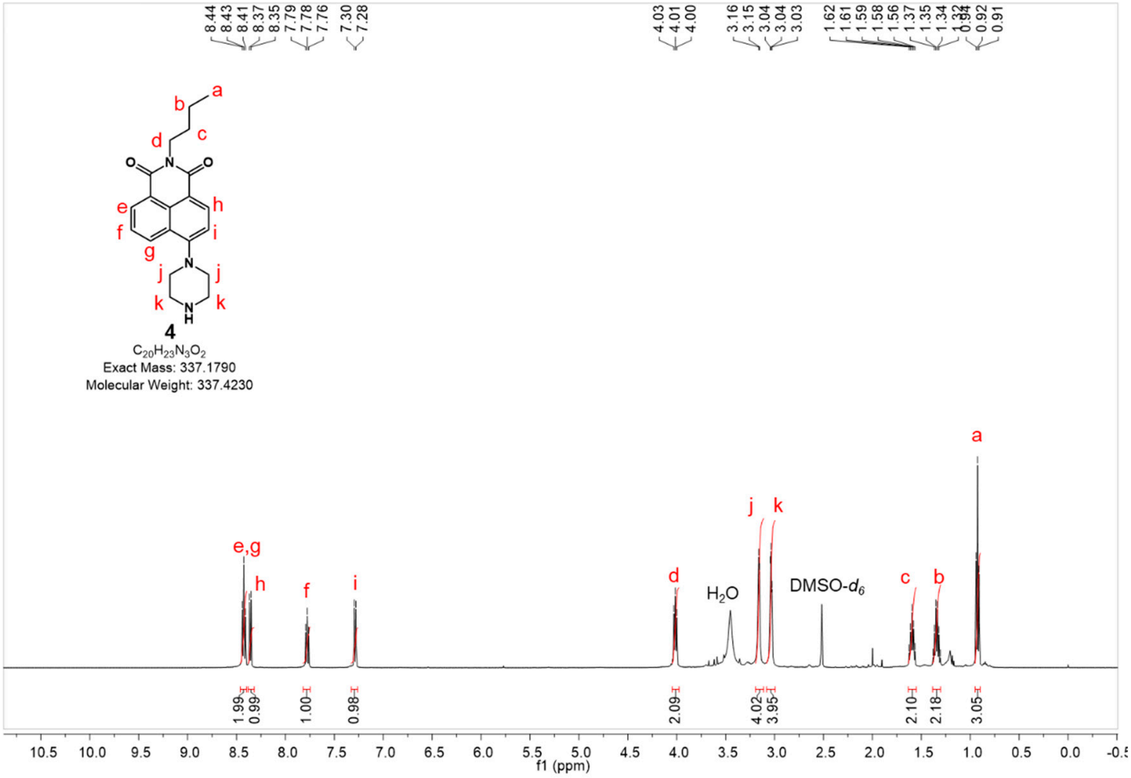


**Figure S17.** $^{1}H$ NMR spectrum of **4** in DMSO-$d_6$.

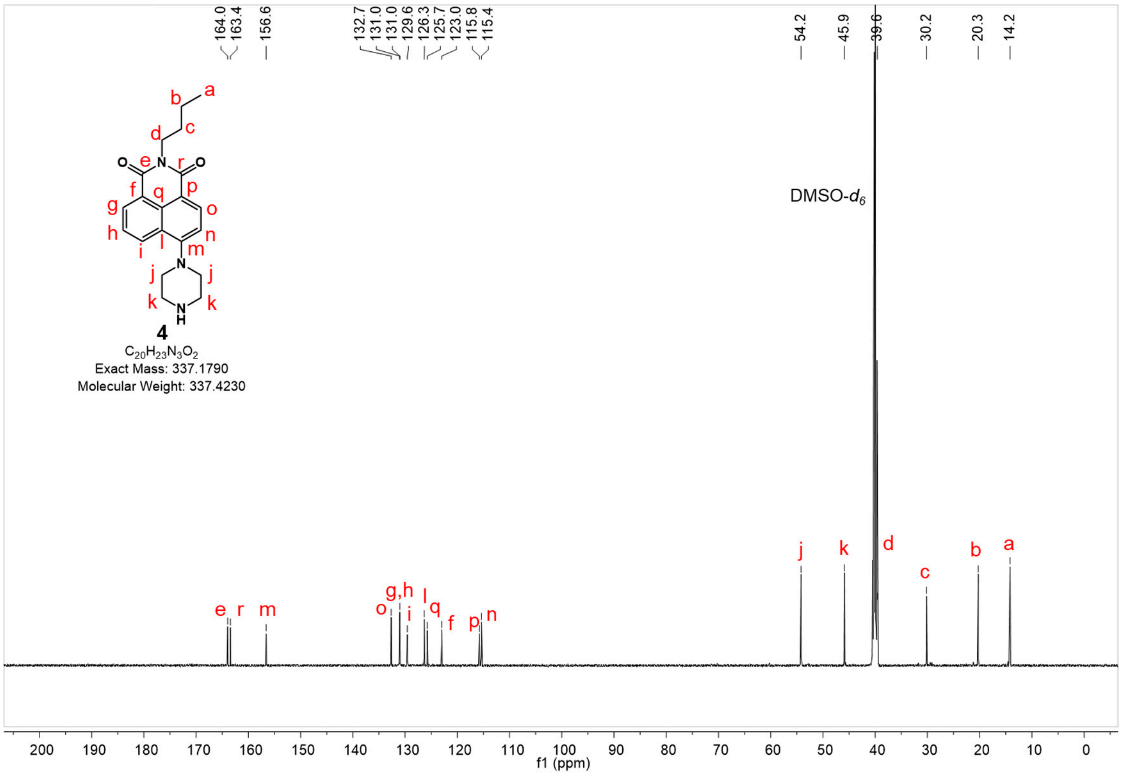


**Figure S18.** $^{13}C$ NMR spectrum of **4** in DMSO-$d_6$.

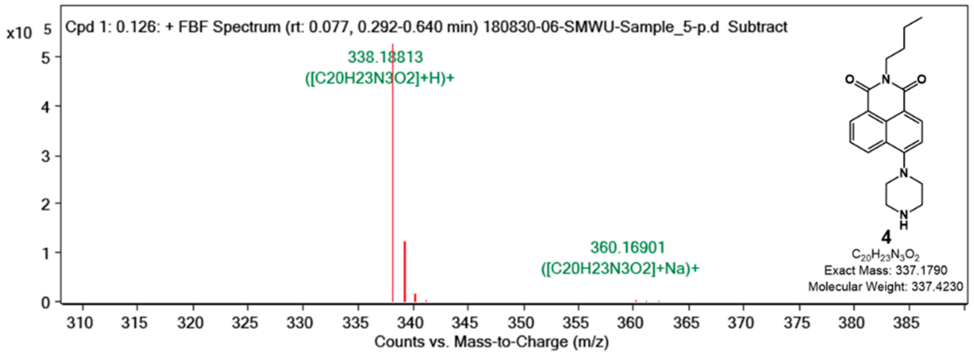


**Figure S19.** HR-ESI-MS spectrum of **4**.

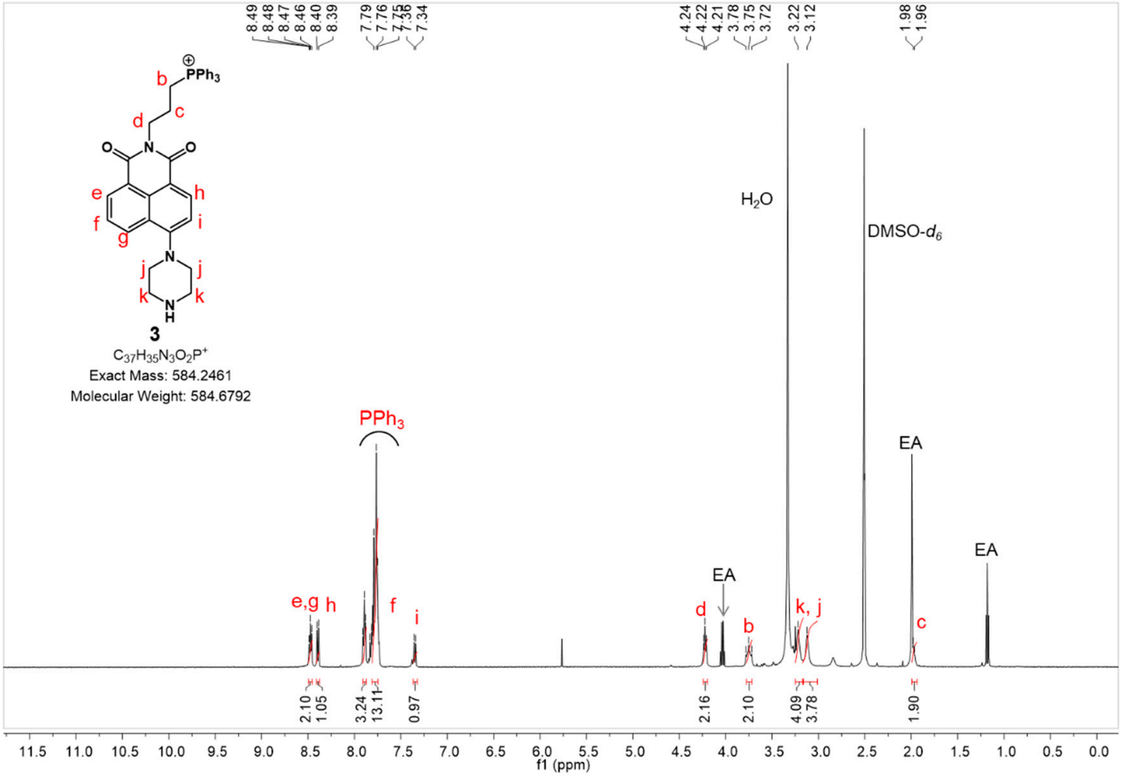


**Figure S20.** $^{1}$H NMR spectrum of **3** in DMSO-$d_6$.

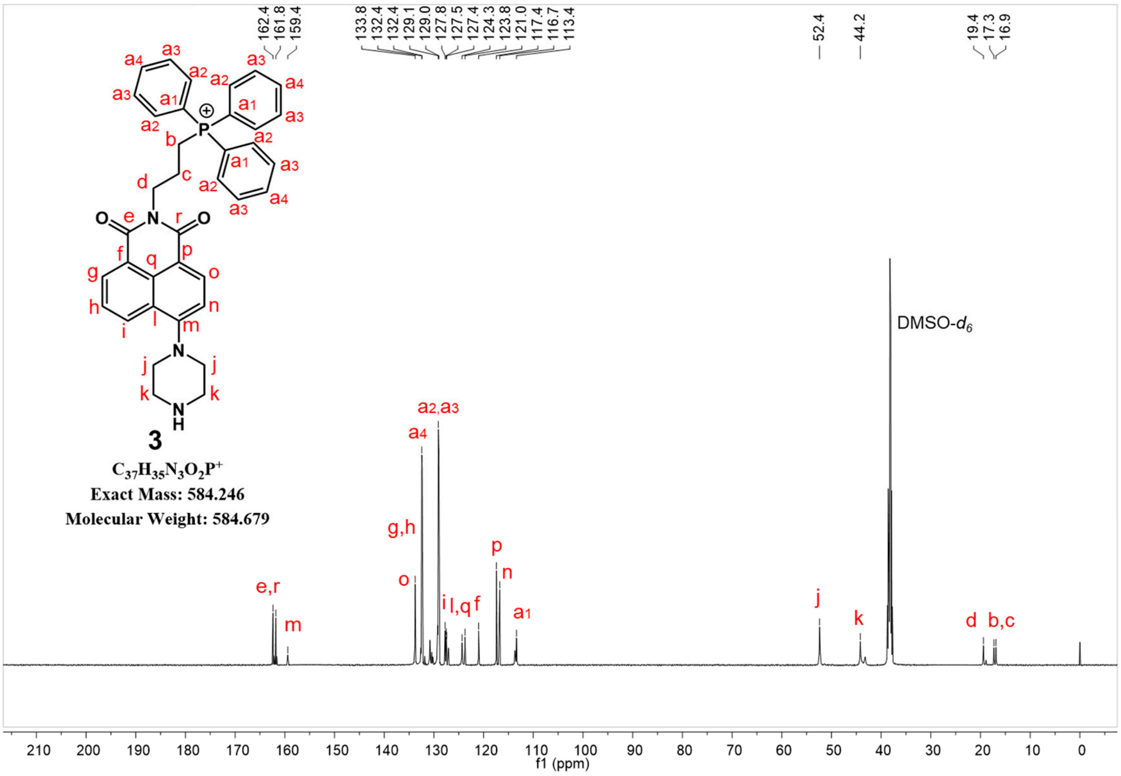


**Figure S21.** $^{13}$C NMR spectrum of **3** in DMSO-$d_6$.

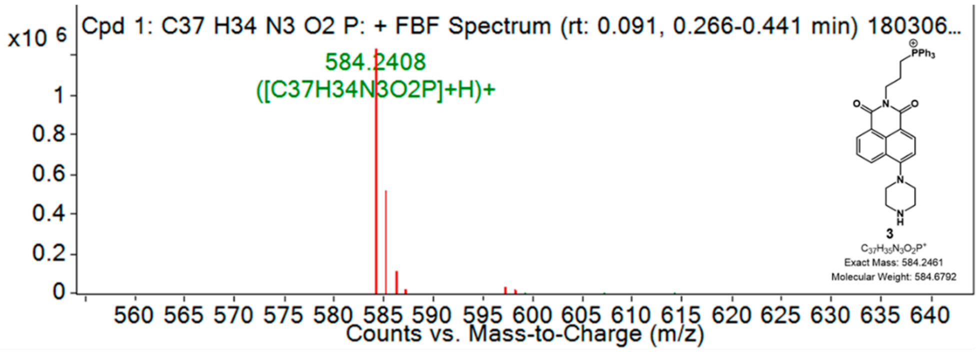


**Figure S22.** HR-ESI-MS spectrum of **3**.

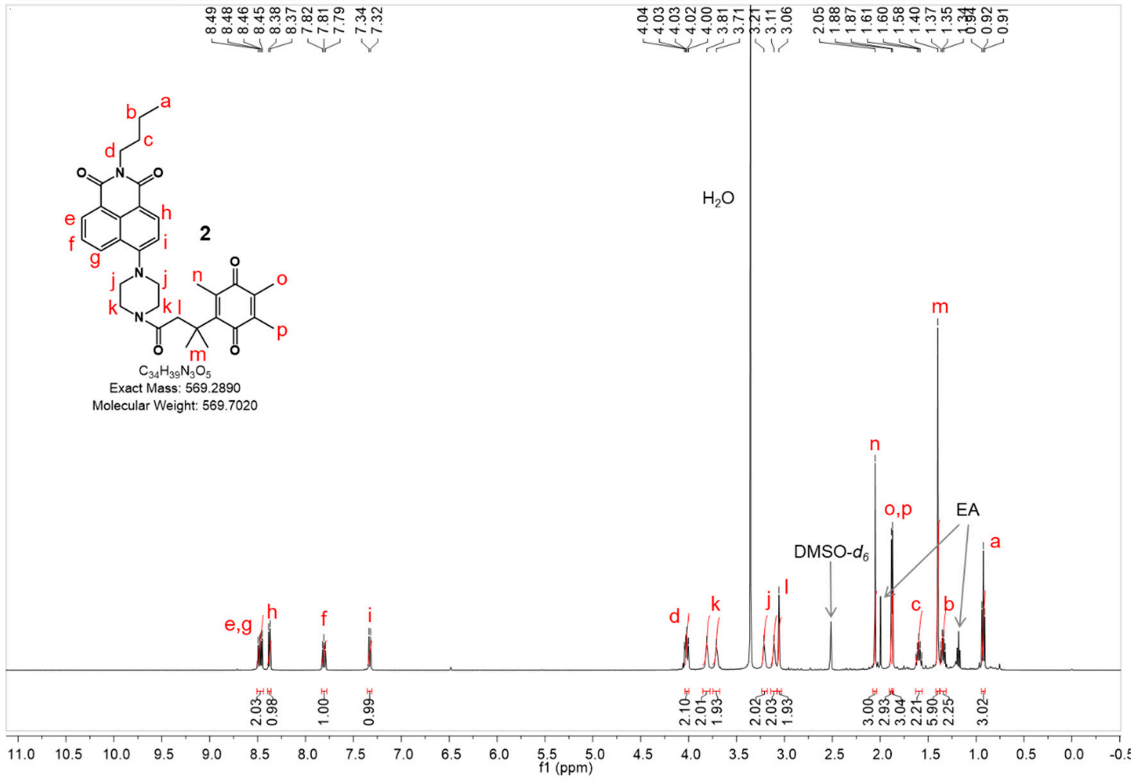


**Figure S23.** $^{1}$H NMR spectrum of **2** in DMSO-*d6*.

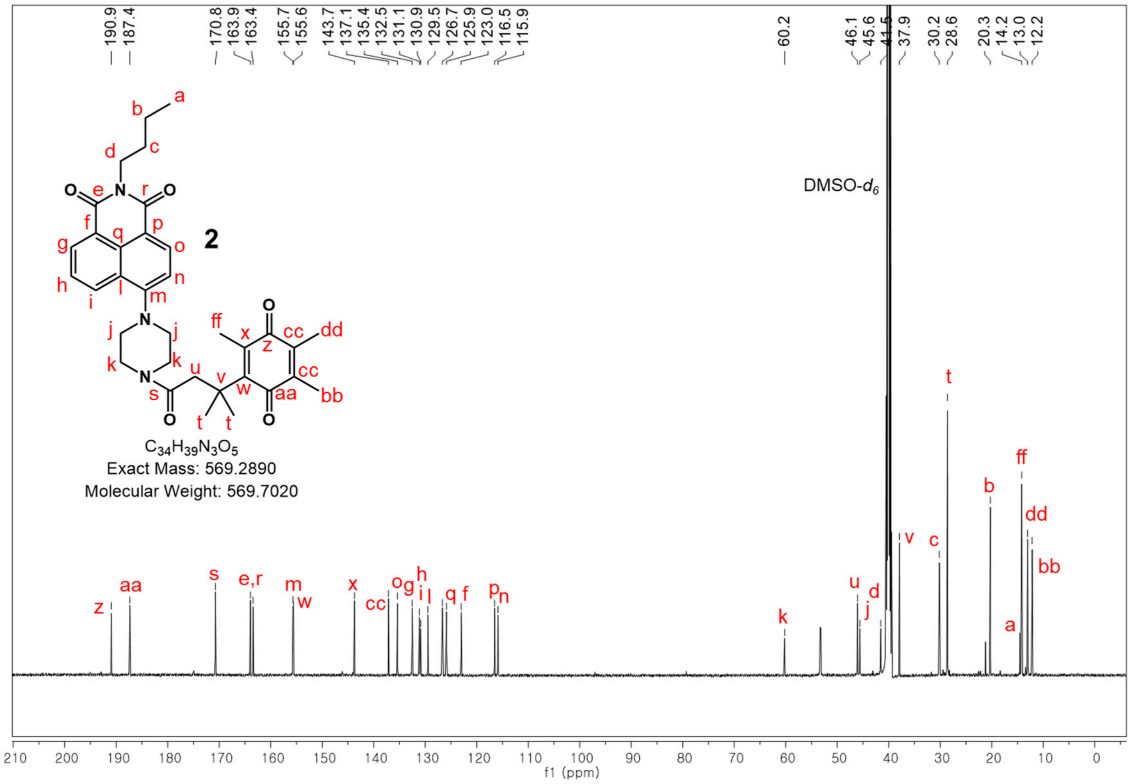


**Figure S24.** $^{13}$C NMR spectrum of **2** in DMSO-*d6*.

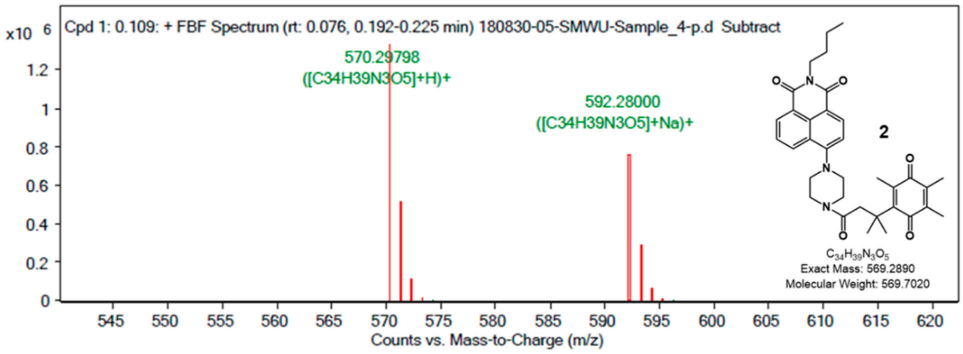


**Figure S25.** HR-ESI-MS spectra of **2**.

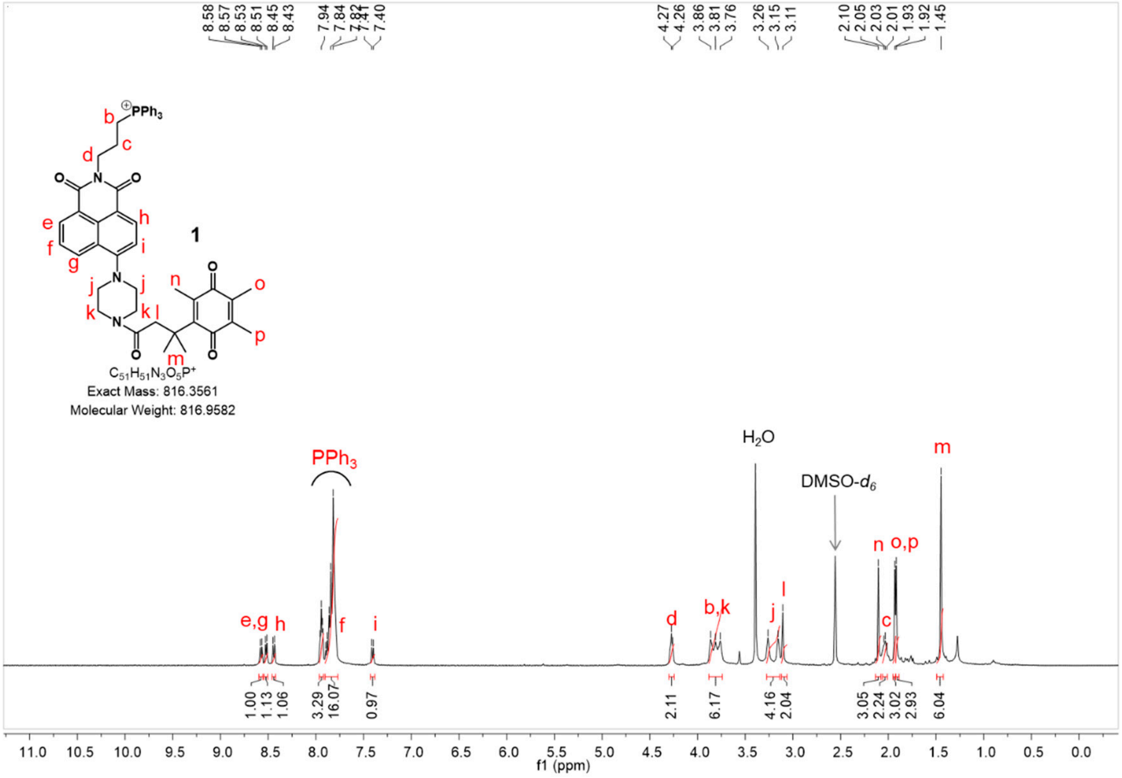


**Figure S26.** $^1$H NMR spectrum of **1** in DMSO-$d_6$.

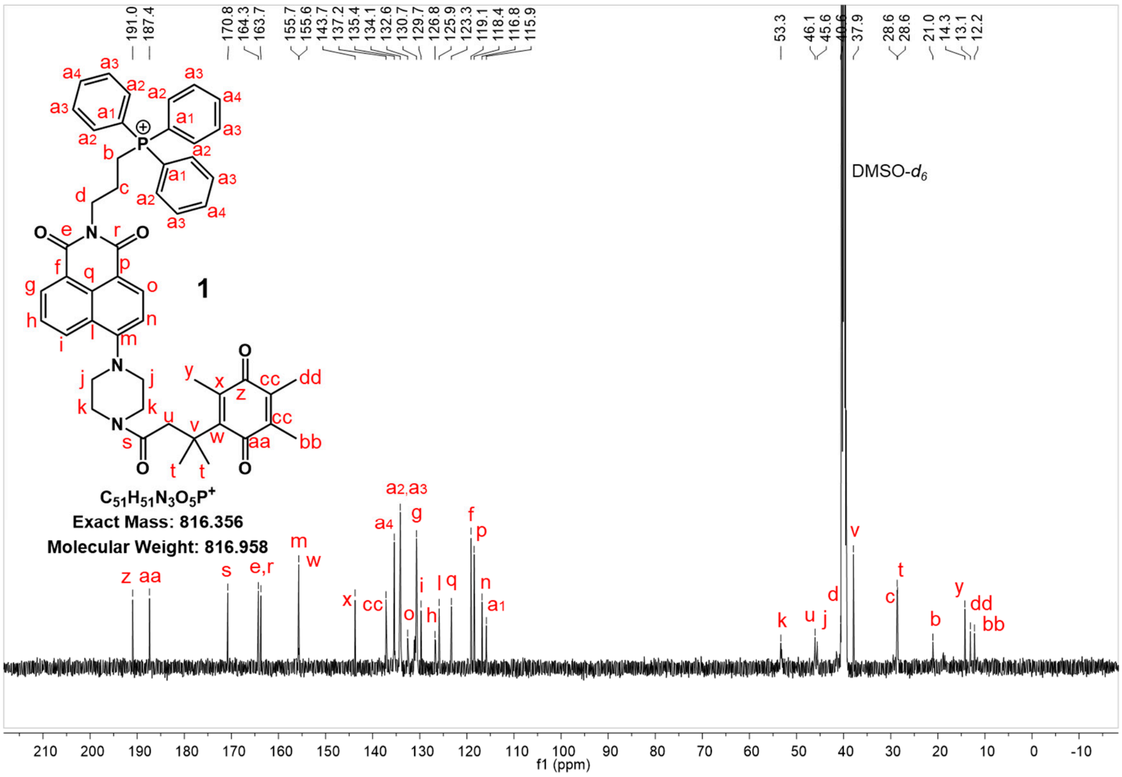


**Figure S27.** $^{13}$C NMR spectrum of **1** in DMSO-*d*$_6$.

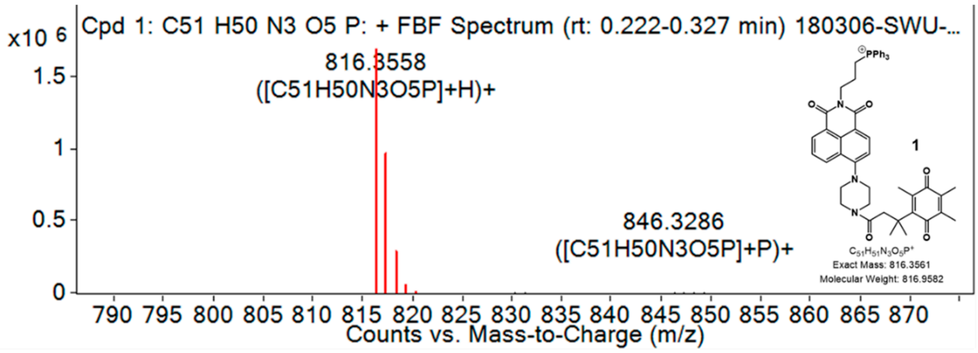


**Figure S28.** HR-ESI-MS spectrum of **1**.
